# Editorial: Biomaterials for dental caries prevention and management

**DOI:** 10.3389/froh.2026.1845714

**Published:** 2026-04-21

**Authors:** Rahena Akhter, Noriko Hiraishi, Kittisak Sanon, Ollie Yiru Yu

**Affiliations:** 1 Sydney Dental School, Faculty of Medicine and Health, The University of Sydney, Sydney, NSW, Australia; 2 Department of Cariology and Operative Dentistry, Graduate School of Medical and Dental Sciences, Institute of Science Tokyo, Tokyo, Japan; 3 Smart Innovations for Preventive and Restorative Dentistry Research Group, Department of Operative Dentistry, Faculty of Dentistry, Chulalongkorn University, Bankok, Thailand; 4 Faculty of Dentistry, The University of Hong Kong SAR, Hong Kong SAR, China

**Keywords:** demineralisation, dental caries, glass ionomer cement, prevention, remineralisation, restorative materials

Dental caries remains a prevalent oral disease and continues to be a major cause of loss of dental hard tissues, restoration failure, and pulpal involvement. Despite advances in preventive strategies and restorative techniques, untreated caries remains the most prevalent oral condition worldwide (1). Restorative treatment is not a permanent solution for dental caries; recurrent caries remain a frequent clinical challenge (2). The Research Topic *Biomaterials for Dental Caries Prevention and Management* was developed to highlight current research directions in the development and evaluation of bioactive dental materials aimed at preventing caries initiation, limiting biofilm development, enhancing remineralization, and supporting tissue repair. The collection brings together original research articles and a systematic review that reflect ongoing efforts to improve the biological performance of preventive and therapeutic materials used in caries management.

A study in this Research Topic addresses the interaction between dental materials and early biofilm formation, which is a critical step in the development of caries lesions. The study investigating cerium chloride pretreatment of hydroxyapatite surfaces examines how surface modification by cerium influences initial bacterial attachment. Using a multi-species biofilm model and surface characterization techniques, this work demonstrates that cerium incorporation and the formation of surface precipitates are associated with reduced early biofilm presence compared with untreated and chlorhexidine-treated controls. This study contributes to the growing body of research exploring alternative ions and surface modifications as strategies to influence microbial colonization on dental hard tissues and biomaterial surfaces (Gade et al.).

Glass ionomer cements (GICs) remain widely used in caries management due to their fluoride release and chemical adhesion to tooth structure (2). However, their mechanical limitations restrict their application in stress-bearing areas (3). Two original research articles in this Research Topic focus on modifying conventional GICs to improve their performance while maintaining bioactivity. One study evaluates the incorporation of microchitosan derived from *Xylotrupes gideon* into GIC. The findings indicate that low concentrations of microchitosan can improve compressive strength, tensile strength, and surface hardness of the material after storage in artificial saliva. The use of insect-derived chitosan also highlights interest in alternative and potentially more sustainable sources of biomaterials. This work provides data supporting the feasibility of bio-polymer reinforcement of conventional GIC formulations (Tjandrawinata et al.).

Complementing this chitosan-based reinforcement approach, another study investigates the effect of reducing glass powder particle size on the physicochemical and mechanical properties of GIC. By producing submicron, nano-sized, and hybrid particle distributions, the authors demonstrate that particle size reduction increases ion release and alkalinity over time, while also affecting setting time and mechanical strength. The hybrid formulation, combining nano- and submicron-sized particles, showed a balance between ion release and acceptable mechanical properties. These findings illustrate the influence of microstructural design on the functional behavior of restorative materials and highlight the trade-offs between bioactivity and mechanical performance that must be considered in material development (Tuygunov et al.).

The management of deep carious lesions and pulp exposure represents another area where biomaterial performance is critical. The development and evaluation of nano *α*-tricalcium phosphate and silver nanoparticle biocomposites for direct pulp capping are presented in this Research Topic. The study reports that selected formulations exhibit sustained calcium and phosphate ion release, stable alkaline pH, and antibacterial activity against cariogenic bacteria. Structural characterization confirmed that incorporation of silver nanoparticles did not alter the crystalline structure of the calcium phosphate matrix. The findings support further investigation of multifunctional pulp capping materials that combine bioactivity with antibacterial properties for applications in vital pulp therapy (Wulansari et al.).

A systematic review included in this Research Topic focuses on the antimicrobial potential of bioactive resin composites in caries management. Secondary caries remains a leading cause of restoration failure, and surface-associated biofilms play a central role in this process. The review summarizes *in vitro* evidence comparing microbial adhesion and antimicrobial activity of bioactive resin composites with conventional resin-based materials. The findings indicate that while bacterial adhesion may be comparable between material types due to similar surface characteristics, bioactive resin composites demonstrate greater antimicrobial effects, likely related to ion release and changes in the local microenvironment (Lopes et al.).

The relevance of bioactive restorative materials in elderly population is addressed in an *in vitro* study evaluating calcium, phosphate, and fluoride ion release from restorative materials. Root caries and cervical lesions are increasingly prevalent in aging individuals, and restorative materials used in these contexts are exposed to varying pH levels and temperature conditions. The study demonstrates that fluoride and calcium ion release from restorative materials is influenced by environmental conditions, material composition, and exposure duration. Material-dependent variation in ion release profiles was indicated, underscoring the importance of considering clinical context and patient-related factors in the selection of restorative materials for caries management in older adults (Aliberti et al.).

Collectively, the articles in this Research Topic reflect current research directions in dental biomaterials, including surface modification to influence biofilm formation, microstructural and compositional modification of restorative materials to enhance ion release and mechanical performance, and the development of multifunctional materials for pulp therapy. While the studies provide important *in vitro* evidence supporting the biological potential of emerging biomaterials, further work is required to establish long-term performance, biocompatibility, and clinical effectiveness. Standardized testing protocols and well-designed *in vivo* and clinical studies will be necessary to support translation into routine clinical practice.

This research topic provides an overview of contemporary approaches to the development and evaluation of biomaterials for dental caries prevention and management. The contributions highlight the continued evolution of preventive and therapeutic materials toward systems that combine mechanical function with biologically relevant properties aimed at controlling caries processes and supporting hard tissue health.

## References

[1] Li X Li R Wang H Yang Z Liu Y Li X Global burden of dental caries from 1990 to 2021 and future projections. Int Dent J. (2025) 75(5):100904. 10.1016/j.identj.2025.100904 40714315 PMC12318333

[2] Ge KX Quock R Chu CH Yu OY. The preventive effect of glass ionomer cement restorations on secondary caries formation: a systematic review and meta-analysis. Dent Mater. (2023) 39(12):e1–e17. 10.1016/j.dental.2023.10.008 37838608

[3] Mahfouz RA Abd El Rahman AM Hanno AG Attia MH. Stress distribution in zirconia-reinforced glass ionomer restorations in molar incisor hypomineralization: a finite element analysis. Dent Mater. (2025) 41(5):607–20. 10.1016/j.dental.2025.03.007 40158933

